# Smoltification of Atlantic Salmon (*Salmo salar* L.) Is Associated with Enhanced Traffic and Renewal of B Cell Repertoire

**DOI:** 10.3390/genes15091220

**Published:** 2024-09-18

**Authors:** Aleksei Krasnov, Sergey Afanasyev, Marianne H. S. Hansen, Marta Bou, Lene Sveen, Jens-Erik Dessen

**Affiliations:** 1 The Norwegian Institute of Aquaculture, Nofima, 9291 Tromsø, Norway; marianne.h.s.hansen@nofima.no (M.H.S.H.); marta.bou@nofima.no (M.B.); lene.sveen@nofima.no (L.S.); jens-erik.dessen@nofima.no (J.-E.D.); 2 Sechenov Institute of Evolutionary Physiology and Biochemistry, 194233 St. Petersburg, Russia; afanserg@mail.ru

**Keywords:** atlantic salmon, B cell traffic, gene expression, IgM sequencing

## Abstract

The smoltification of farmed Atlantic salmon is commonly associated with mild immunosuppression. However, B cells may deviate from this trend, showing increased proliferation and migration during this period. This study assessed the effects of smoltification and adaptation to seawater in a controlled experiment. Analyses were conducted on the head kidney, spleen, gill, and both visceral and subcutaneous fat (VAT, SAT) across four time points: parr, early and complete smoltification, and twelve weeks post-seawater transfer. Gene expression analysis was performed to track the distribution and developmental changes in their B cells. Expression profiles of three types of immunoglobulins (*ig*), including membrane-bound and secreted forms of *igm*, as well as B cell-specific markers *pax1* and *cd79*, showed strong correlations and contrasted with profiles of other immune cell markers. The highest levels of expression were observed in the lymphatic tissue, followed by the VAT. Enhanced expression in the gill and adipose tissues of smolts suggested an increase in B cell populations. Parallel sequencing of the variable region of the IgM heavy chain was used to track B cell traffic, assessed by the co-occurrence of the most abundant sequences (clonotypes) across different tissues. Smoltification markedly enhanced traffic between all tissues, which returned to initial levels after twelve weeks in the sea. The preferred migration between the head kidney, spleen, and VAT supports the role of abdominal fat as a reservoir of lymphocytes. These findings are discussed in the context of recent studies that suggested the functional significance of B cell traffic in Atlantic salmon. Specifically, the migration of B cells expressing secreted immunoglobulins to virus-infected hearts has been identified as a key factor in the disease recovery and survival of fish challenged with salmon alphavirus (SAV); this process is accelerated by vaccination. Additionally, the study of melanized foci in the skeletal muscles revealed an association between antigen-dependent differentiation and the migration of B cells, indicating a transfer from local to systemic immune responses. Updating the antibody repertoire in the lymphatic and peripheral tissues of smolts may assist in their adaptation to the marine environment and in encountering new pathogens. Emerging evidence highlights B cell migration as an important and previously unrecognized immune mechanism in salmonids.

## 1. Introduction

The life cycle of Atlantic salmon includes freshwater and seawater phases. Their anadromous migration from the river to the sea is preceded by a complex developmental process known as smoltification. Dramatic endocrine changes, leading to increased levels of pituitary hormones and corticosteroids, affect salmon morphology, metabolism, osmoregulation, sensory system, and behavior, as reviewed in [1,2]. The smoltification of farmed Atlantic salmon is associated with moderate immune suppression, which may persist for at least several months after seawater transfer. Transcriptome analyses have revealed this trend in several studies performed at different research stations and farms [3,4,5]. However, two published studies did not observe immune suppression [6,7], and the number and composition of downregulated genes varied among studies. The inconsistent nature of immune changes in smolts suggests that these alterations likely develop as a side effect of complex endocrine regulation, making high throughput analyses necessary for their detection.

A notable exception to the trend in immune suppression during smoltification is the apparent increase in B cell activity. An increased abundance of immunoglobulin (Ig) transcripts was observed as early as the first study that identified the impact of smoltification on the immune system [3]. A longitudinal field survey of two cohorts of Atlantic salmon produced at commercial farms found an increase in total immunoglobulin M (IgM), circulating heterologous antibodies, and vaccine-induced bacteria-specific antibodies in smolts, followed by a gradual decrease after seawater transfer [8]. Sequencing of the variable region of the IgM heavy chain (Ig-seq) [9] has made it possible to investigate a previously unknown aspect of B cell activity: directed migration between tissues. The Ig heavy chain variable region is formed by the somatic recombination of the V, D, and J genes, coupled with enzymatic insertions and deletions. The likelihood of two identical sequences appearing independently in different tissues is low, especially considering that early B cell differentiation is likely limited to the lymphatic organs. Therefore, the presence of identical transcripts in different tissues is generally explained by B cell trafficking. Ig-seq performed in this field study suggested that B cell migration is stimulated in smolts, mirroring the profiles of plasma IgM and antibodies. Here, we report the results of a controlled experiment that explored the effects of smoltification and seawater transfer using Ig-seq and RT-qPCR analyses of B cell-specific gene expression, supplemented with several gene markers of other immune cells. Analyses were conducted on parr and smolts in freshwater and after seawater transfer. The head kidney and spleen were included in the analyses as lymphatic tissues. The gill was chosen as a preferred tissue to monitor the interaction between the immune system of Atlantic salmon and the environment [10]. A recently published study [11] and transcriptome analysis indicated that visceral fat (VAT) in Atlantic salmon serves as a depot for lymphocytes, while subcutaneous fat (SAT) is characterized by low immune activity. The functional significance of B cell trafficking is discussed in the context of this and previous studies.

## 2. Materials and Methods

### 2.1. Fish

Analyses were performed on Atlantic salmon from a trial reported in [12]. All operations with fish were authorized by the Norwegian Food Safety Authority (FOTS) under general husbandry practices and ID24383. This study used fish from the group that was kept at a constant temperature of 12 °C and received a commercial feed. The fish were reared in a freshwater flow-through system (Tromsø Aquaculture Research Station, Kårvika, Norway) at dissolved oxygen > 85% saturation, continuous feeding, and light-dark (LD) photoperiod 24:00 until they reached a weight of 18.8 ± 2.3 g. Smoltification was induced by light manipulation using a square wave photoperiod regime including six weeks of winter signal (LD 12:12) followed by eight weeks of constant light, LD 24:00. This method is standard practice in commercial Atlantic salmon aquaculture. Smoltification was assessed using a seawater challenge test [13] and Na-K ATPase activity assay in the gill [14]. After this 14-week period, the fish were moved to seawater flow-through tanks and the trial continued for the next 12 weeks. Fish were sampled at weeks 0, 3, 6, 8, 14, 20, and 26. In this study, analyses were performed on four time points: T1—parr (week 0); T4—presmolt or smolt-1 (week 8); T5—smolt-2 (week 14); and T7—12 weeks in seawater (week 26). At T4, salmon were able to cope with the seawater challenge in contrast to fish tested at previous time points (T3, week 6). However, Na-K ATPase activity was lower than at subsequent T5 (14 weeks), indicating incomplete smoltification. At each time point, six fish were euthanized using a bath overdose of Benzocaine (Benzoak vet, 200 mg/mL, EuroPharma, Leknes, Norway) and were sampled. The head kidney, spleen, gill, VAT, and SAT were stored in RNALater (Thermo Fisher Scientific, Waltham, MA, USA) at −20 °C.

### 2.2. RNA Extraction, Gene Expression

Small tissue samples (5–10 mg) were placed in tubes containing 400 µL of lysis buffer (Qiagen, Düsseldorf, Germany), proteinase K (50 mg/mL), and beads. The samples were homogenized using a FastPrep 96 (MP Biomedicals, Eschwege, Germany) for 120 s at maximum shaking speed, then centrifuged and incubated at 37 °C for 30 min. RNA was extracted using the Biomek 4000 robot (Beckman Coulter, CA, USA) with the Agencourt RNAdvance Tissue kit. RNA concentration was measured with a NanoDrop One (Thermo Fisher Scientific, Waltham, MA, USA), and quality was assessed using a Bioanalyzer 2100 (Agilent, Santa Clara, CA, USA). The RNA was treated with DNase I (Thermo Fisher Scientific, Waltham, MA, USA), and cDNA was synthesized using the TaqMan Reverse Transcription Reagent (Applied Biosystems, Waltham, MA, USA) with random hexamers. RT qPCR analyses included three types of immunoglobulins: *igd*, *igt*, and membrane-bound and secreted forms of *igm* (*migm* and *sigm*). The transcription factor *pax5* plays an essential role in B cell differentiation [15] and *cd79* is a part of the B cell receptor [16]. *Cd83* and *cd40* were included as markers of professional antigen-presenting cells [17,18]. *Cd28* is a co-stimulatory receptor of T cells. Primer sequences are provided in [11]. *Ef1a* was selected as a reference gene due to homogenous distribution in Atlantic salmon tissues. PCR was run in a QuantStudio5 real-time quantitative PCR system (Applied Biosystems, Waltham, MA, USA), and the reaction mixture contained 4 µL (21 ng/µL) of diluted cDNA, 5 µL SYBR™ Green Master Mix (Applied Biosystems, Waltham, MA, USA), and 1 µL of the forward and reverse primer. The program included heating for 1 min at 95 °C, amplification (1 s at 95 °C, 20 s at 60 °C), and a melting curve stage; the total number of cycles was 40. Each biological sample was run in duplicates for all genes. The −ΔCt values were calculated subtracting the Ct values of the reference gene. The data were centered by adding the mean value of each gene in the entire dataset to the corresponding data points, ensuring that the mean of the data was zero.

### 2.3. IgM Repertoire Sequencing

The CDR3 region or V(D)V junction of *igmhc* was sequenced. The cDNA synthesis was carried out using SuperScript IV reverse transcriptase (Thermo Fisher Scientific, Waltham, MA, USA) and oligonucleotides aligning to the constant region (CH) of Atlantic salmon *igm*. (TAAAGAGACGGGTGCTGCAG). Libraries for sequencing were prepared with two PCR reactions. The first PCR-amplified cDNA contained a degenerate primer, TCGTCGGCAGCGTCAGATGTGTATAAGAGACAGTGARGACWCWGCWGTGTATTAYTGTG, which aligns to the 3′-end of all Atlantic salmon VH genes, and a primer GTCTCGTGGGCTCGGAGATGTGTATAAGAGACAGGGAACAAAGTCGGAGCAGTTGATGA to the 5′-end of CH. Both primers were designed to match Illumina Nextera adaptors. The reaction mixture (20 µL) included 10 µL 2X Platinum™ Hot Start PCR Master Mix (Thermo Fisher Scientific, Waltham, MA, USA), 0.5 µL of each primer (10 pmol/µL), 8 µL water, and 1 µL template. The second PCR used Nextera™ XT Index Kit v2 (Illumina, San Diego, CA, USA), and the reaction included 2 µL of each primer and 2 µL product from the first PCR. The PCR program included the following steps: initial heating for 1 min at 94 °C, followed by amplification for 10 s at 94 °C, 20 s at 53 °C, and 20 s at 72 °C (30 cycles in the first PCR and 9 cycles in the second PCR), with a final extension of 5 min at 72 °C. The DNA concentration of the amplified product was measured using Qubit (Thermo Fisher Scientific, Waltham, MA, USA). Aliquots of the libraries were combined and purified twice using a PCR clean-up kit (Qiagen, Düsseldorf, Germany). Sequencing was carried out using the Illumina MiSeq™ Reagent Kit v3, 150 cycles (Illumina, San Diego, CA, USA). Libraries were diluted to 4 nM, and a PhiX control was added at a final concentration of 0.8 nM. After trimming the Illumina adaptors and primers and removing low-quality reads, the sequences were transferred to a relational database where the frequency of each unique sequence (clonotype) was evaluated. The trafficking of B cells was assessed through pairwise comparisons of tissues. The 100 largest clonotypes were selected, and their occurrence in a second tissue was evaluated at a threshold frequency greater than 2 × 10^−5^.

### 2.4. Statistics

Data were analyzed with ANOVA, followed by a post hoc Tukey test (*p* < 0.05) using Statistica 14.01.25 (TIBCO, Palo Alto, CA, USA). Normality was assessed using the Kolmogorov–Smirnov test. The same program was used for correlation analysis and hierarchical clustering.

## 3. Results

### 3.1. Gene Expression: Differences between Tissues and Temporal Changes

**Figure 1:**
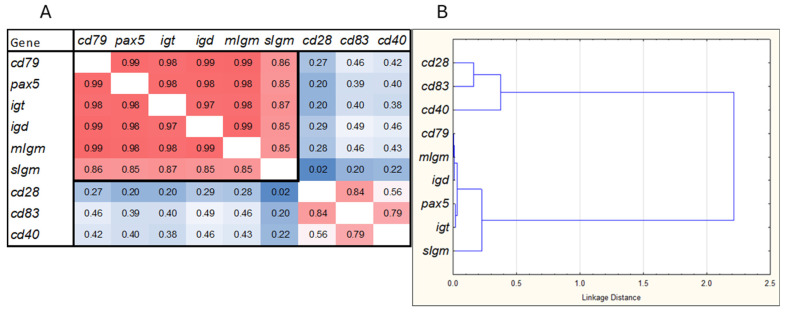
Relationship between gene expression profiles in the entire dataset. (**A**): Correlation (Pearson r). The border line delimits B cell-specific genes. (**B**): Hierarchical clustering (Pearson r, Ward’s method).

**Figure 2:**
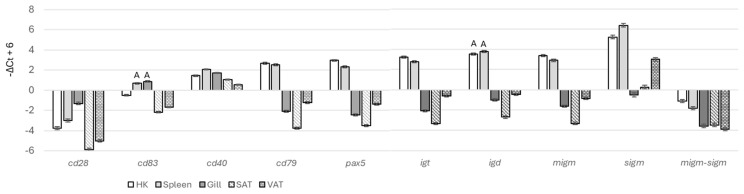
Tissue expression of immune genes. Data (−ΔCt) were centered as described in Section 2.2. *Migm–sigm* presents differences between secreted and membrane isoforms of *igm*. Bars not sharing common letters are significantly different (ANOVA, Tukey test, *p* < 0.05).

**Figure 3:**
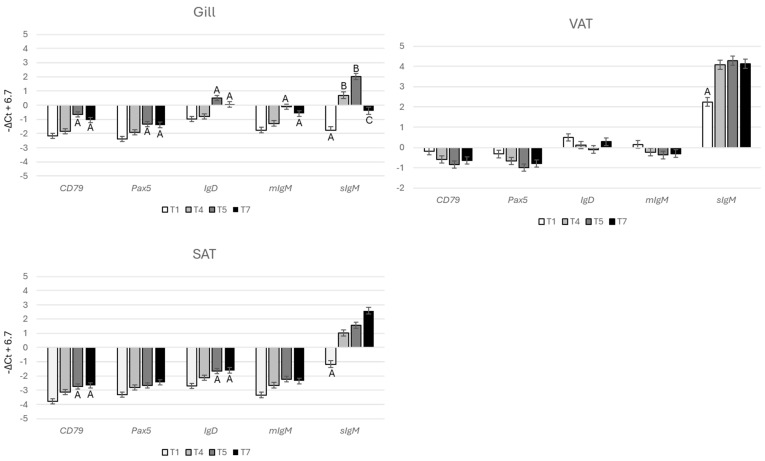
Expression of B cell-specific genes across tissues and time points. Data (−ΔCt) were centered as described in Section 2.2. Bars that do not share common letters are significantly different (ANOVA, Tukey test, *p* < 0.05).

The expression profiles of all B cell-specific genes (*cd79*, *pax5*, *igt*, *igd*, *migm*, and *sigm*) exhibited strong correlations (Figure 1A), with a Pearson correlation coefficient of r = 0.94 ± 0.06 (mean ± SD). These genes formed a dense cluster, with *sigm* showing some separation from the other four genes (Figure 1B). The lower correlation between *sigm* and the other B cell markers is likely due to the presence of antibody-producing plasma cells, which express the isoform-encoding secreted antibodies at much higher levels compared to *pax5*, a regulator of differentiation, and *cd79*, a component of the B cell receptor. For comparison with B cells, three genes associated with other specialized immune cells (*cd28*, *cd83*, and *cd40*) were included in the analysis. As expected, these genes did not show a strong correlation with B cell markers. The expression patterns were unique to each tissue, and even seemingly small differences were significant (Figure 2). The expression of B cell-specific genes was markedly higher in lymphatic tissues. *Igd* levels were similar in both the head kidney and spleen. However, *igt* and *migm* expression was higher in the head kidney, while *sigm* was upregulated in the spleen, which is considered a secondary lymphatic organ in teleost fish [19]. Overall, the secreted isoform constituted the majority of *igm*, with the *sigm*-to-*migm* ratio being higher in the peripheral tissues, indicating a predominance of antibody-producing cells. While most B cells reside in specialized immune organs, the tissue distribution of other immune cells can be less polarized. For instance, *cd28* expression was highest in the gill, while *cd83* expression was highest in both the gill and spleen. B cell-specific genes did not show significant temporal changes in the head kidney and spleen. However, *sigm* expression increased in the gill, VAT, and SAT during the smolt-1 stage (T4, Figure 3) and decreased in the gill by the end of the trial. The expression of other B cell-specific genes increased in the gill at the end of smoltification (T5), while adipose tissue showed smaller changes.

### 3.2. Sequencing of the Variable Region of igm Heavy Chain (Ig-seq) Reveals B Cell Migration

**Figure 4:**
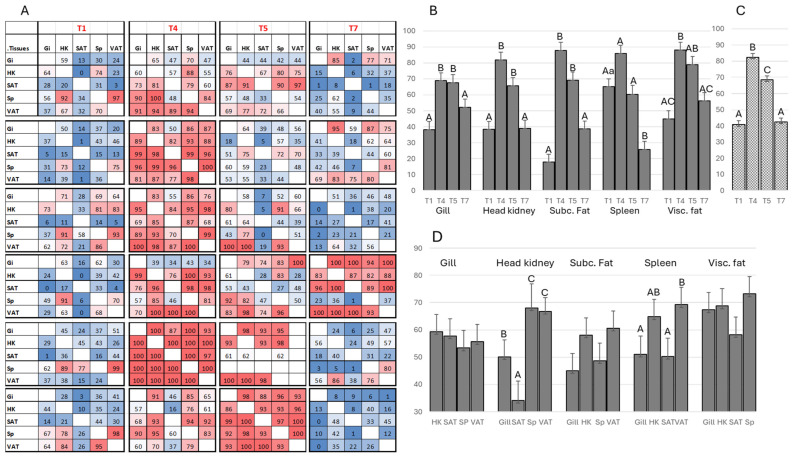
IgM sequencing. B cell migration assessed by co-occurrence of largest clonotypes. Tissues were compared in pairs to determine how many of the largest hundred clonotypes in the first tissue were present in the second tissue. (**A**): Individual data, six fish were analyzed at each of the four time points. The number in each square means the amount of clonotypes detected in both tissues. (**B**,**C**): Temporal changes, average numbers of clonotypes in each tissue shared with all other tissues at every time point (**B**) and number of shared clonotypes in all tissues across each time point (**C**). Bars not sharing common letters are significantly different (ANOVA, Tukey test, *p* < 0.05). Capital and lowercase letters denote time points and tissues, respectively. (**D**): Overlap between tissues, averaged across all time points. Bars not sharing common letters are significantly different (ANOVA, Tukey test, *p* < 0.05).

The migration of B cells is assessed by the co-occurrence of the largest clonotypes in the compared tissues. Given the nearly unlimited sequence variation in heavy chain CDR3, the presence of identical sequences in the variable regions of two tissues is primarily explained by the movement of expanded B cells. We use an asymmetric metric that prioritizes the largest clonotypes, which are likely to include recently expanded clones. In each comparison, the hundred largest clonotypes are selected from the first tissue, and their presence is checked in the second tissue; the values range from zero to one hundred. The tissues are then reversed for comparison. Both individual (Figure 4A) and grouped data (Figure 4B,C) clearly show B cell migration associated with smoltification, returning to initial levels after three months in seawater. The low co-occurrence of clonotypes in all tissues of parr was followed by a dramatic increase in all presmolts sampled in the middle of the constant light period. The results indicated the active migration of B cells in two fish at T5 and one fish at T7. The temporal profiles were similar across all tissues, with certain differences. Unlike other tissues, the spleen showed high co-occurrence of the largest clonotypes already in parr, with subsequent increases being insignificant. Peak levels in smolts were followed by a decrease at the last time point. The tendency to decrease at T4 was noticed in all tissues, and a significant difference between T4 and T5 was observed when pooled data were analyzed (Figure 4A). The reduction after 12 weeks in seawater was significant in all tissues except visceral fat. The directions of traffic were compared (Figure 4D). Peripheral tissues showed equal sharing of the largest clonotypes with other tissues; the mean values were highest in visceral fat and lowest in subcutaneous fat. Preferred migration was observed in the lymphatic tissues. Both the head kidney and spleen showed the highest match with each other and with visceral fat, while migration to the gill and subcutaneous fat was weaker.

## 4. Discussion

The humoral adaptive immune system of teleost fish is characterized by its relatively low affinity for antibodies and several primitive features, such as an absence of lymph nodes and isotype switching. The presence of germinal center-like structures in the spleen of rainbow trout and somatic hypermutations in the variable region of IgM were recently reported [20]. However, this process occurs on a much smaller scale compared to warm-blooded animals, and it remains uncertain whether hypermutations lead to affinity maturation. Antibody responses in fish develop slowly, especially at low water temperatures, and the duration of vaccine protection is relatively short [21]. Additionally, the existence of memory B cells in fish is still unclear. Nonetheless, B cells play a crucial role in the defense of Atlantic salmon against pathogens. The use of multicomponent vaccines in commercial aquaculture has significantly reduced the morbidity and mortality caused by infectious diseases, as well as the use of antibiotics [22,23,24]. This provides compelling evidence of the importance and effectiveness of antibody responses. The apparent contradiction between the low efficiency and the importance of these defenses can be partly explained by the specific features of fish antibodies and B cells. Fish produce large amounts of heterologous antibodies (also known as natural antibodies), which are detected by their binding to model antigens; their titers are much higher than those of antigen-specific antibodies [25,26]. Vaccination and infection markedly increase both the levels of heterologous antibodies and the range of antigens they recognize [27,28]. These antibodies are likely involved in rapid defense against pathogens, providing a time gain before a more specific and effective antibody response occurs. High-throughput parallel-sequencing of the IgM repertoire has elucidated another aspect of teleost B cells, namely their migration between tissues.

The Ig heavy chain variable region is formed through the somatic recombination of the V, D, and J genes, along with enzymatic insertions and deletions. The likelihood of two identical sequences appearing independently in different tissues is low, especially considering that early B cell differentiation is most likely confined to lymphatic organs. Therefore, the presence of identical CDR3 transcripts in different tissues is generally explained by B cell trafficking. Combining Ig-seq with B cell-specific gene expression may reveal the link between B cell migration and differentiation. Repertoire sequencing has raised two important questions concerning where antibodies are produced and how they spread. It was previously believed that the antigen-dependent differentiation of plasma cells occurs in the lymphatic organs, with antibodies then released into the circulation. However, transcriptomics and repertoire sequencing challenge these widely accepted views in fish immunology. Transcriptomic analysis using oligonucleotide DNA microarrays has shown an association between increased *igm* transcripts in salmon alphavirus (SAV)-infected Atlantic salmon hearts and virus clearance [29,30]. RNA sequencing identified a high abundance of *igm* transcripts as the main difference between dead fish and survivors, with transcripts of the secreted isoform prevailing [31]. Ig-seq demonstrated enhanced trafficking of IgM-expressing B cells to virus-infected hearts, which was accelerated in salmon vaccinated against SAV [28,32]. The targeted delivery of antibodies to infected tissue likely enhances their efficacy, thus mitigating the limitations of low affinity. Evidence of antigen-dependent B cell differentiation followed by active trafficking was obtained in a study of melanized foci in the skeletal muscle of Atlantic salmon [11]. Previously, it was thought that B cells migrate only from the lymphatic to peripheral tissue. However, the local differentiation of B cells suggests that antibody responses can be initiated at each site, and the movement of B cells converts local responses into systemic ones.

Enhanced traffic of B cells in Atlantic salmon smolts has been demonstrated in both field studies and controlled experiments. What benefits can be gained from this? We considered the possible spread of naïve B cells, which could constitute the first line of humoral adaptive immunity in peripheral tissues. The membrane and secreted forms of *igm* (*migm* and *sigm*) are expressed at high levels in naïve and antibody-producing B cells, respectively. A transient increase in *migm* expression was observed in the gills of smolts, but not in adipose tissues. The *migm*-to-*sigm* ratio was consistently higher in the head kidney and spleen and did not change with increased B cell traffic. This suggests that B cells that had already entered or completed antigen-stimulated differentiation were predominant among migrating B cells. Overall, gene expression suggested minor developmental changes throughout the study period. Notably, active traffic from the spleen was observed as early as the first time point (parr), earlier than between other tissues. It is important to note that differentiation waves could occur over short periods and remain undetected due to large sampling intervals. Unlike [11], this study did not observe an association between antigen-dependent differentiation and B cell migration. This issue requires further research with more frequent sampling from the onset of constant light stimulation of smoltification to the end of this period. Did recruited B cells substitute or expand the peripheral populations? The second possibility seems more likely, considering gene expression data. Transcripts of B cell-specific genes increased in all tissues of presmolts, with a further increase in *sigm* observed in the gills of smolts. The ability to recognize novel pathogens and initiate antigen-dependent differentiation and antibody response is determined by the size and diversity of immunoglobulins. Therefore, updating the repertoire during smoltification may help salmon adapt to a marine environment containing many pathogens that they have not been exposed to during their freshwater life period. To date, changes in the IgM repertoire have been demonstrated at the transcriptional level. To determine whether these changes improve salmon’s defense against pathogens at sea, it will be important to assess whether the diversity of antibodies and their ability to recognize and bind to a wider range of antigens increase in smolts.

Adipose tissue was included in the study to learn more about its role in the immune system of Atlantic salmon. VAT transcriptome [33] indicates the presence of large lymphocyte populations. Reports of massive secretions of cytokines [34,35] and infiltration of fat with leukocytes and lymphocytes [36,37] associated with obesity in mammals have stimulated studies of the immune properties of salmonid adipose tissue. There is now more evidence for its importance in adaptive immune responses than for the adverse effects of inflammation. Abdominal fat harbors various cell types, including B cells expressing IgM, IgD, and IgT at different developmental stages. It responds to adjuvants, binds antigens, and mounts antigen-dependent antibody responses [38,39,40]. The IgM-secreting capacity of adipose tissue B cells is markedly higher than that of blood B cells [38]. Changes in the cellular composition of adipose tissue were observed after challenge with viral hemorrhagic septicemia virus. The decreased number of IgM+ cells in parallel with elevated antibody production was interpreted as their differentiation into plasma cells [38]. However, the egress and migration of B cells toward infection foci could also occur. Increased movement between various tissues and visceral fat suggests that this tissue can accumulate B cells that release antibodies into circulation or migrate to other tissues.

## 5. Conclusions

Smoltification prepares salmon for life in the sea by changing their endocrine system, osmoregulation, and behavior. The active migration of B cells, leading to an increase in the size and complexity of the antibody repertoire, may be a new aspect of preadaptation to the new environment. The study adds to the understanding of B cell traffic as an important mechanism of the immune system of salmon and the immune role of adipose tissue. Future research is needed to gain mechanistic insights into the cues driving B cell migration and to provide more direct evidence of its importance for salmon protection against pathogens.

## Data Availability

The raw data supporting the conclusions of this article will be made available by the authors on request.
